# Use of nasal mucosal autonomic nerve response in efficacy evaluation of vidian neurectomy for allergic rhinitis: a prospective study

**DOI:** 10.3389/fnins.2025.1424629

**Published:** 2025-03-25

**Authors:** Linzheng Liu, Na Ma, Yan Niu, Yuping Peng, Yan Li, Jiangjiang Shen, Jiameng He, Jindi Sun

**Affiliations:** ^1^Department of Otolaryngology Head and Neck Surgery, Yulin City First Hospital of Yan'an University, Yulin, China; ^2^Department of Otolaryngology Head and Neck Surgery, Yan'an University, Yan'an, China

**Keywords:** allergic rhinitis, vidian nerve, nasal mucosal autonomic nerve response, sympathetic skin response, waveform

## Abstract

**Objective:**

To evaluate the use of nasal mucosal autonomic nerve responses as an objective indicator for assessing the efficacy of vidian neurectomy (VN) in treating allergic rhinitis (AR).

**Methods:**

Thirty-five patients with moderate to severe AR and 35 healthy controls were included. Autonomic nerve responses were measured before and 1 month after VN surgery, using respiratory stimulation on the nasal mucosa and the opisthenar area. Three waveform types (P-type, N-type and M-type) were identified.

**Results:**

While three waveform types were identified in the nasal mucosa, only the M-type was observed in the opisthenar sympathetic skin response. Preoperative measurements showed higher autonomic responses in patients with AR compared with controls. Following VN, the responses in patients with AR decreased significantly, aligning closely with the control group. No significant changes were observed in the opisthenar responses, indicating a localised effect of VN. Comorbidities such as nasal polyps, sinusitis and deviated septum did not impact the results.

**Conclusion:**

Nasal mucosal autonomic nerve response provides a reliable, objective measure for evaluating the effectiveness of VN in treating AR.

## 1 Introduction

Allergic rhinitis (AR) is an inflammatory disease of the nasal mucosa caused by a variety of genetic and environmental factors (Nevis et al., 2016). It is characterised by symptoms such as nasal congestion, rhinorrhoea, sneezing and nasal itching (Matsumoto et al., 2024). The primary treatments for AR include drug therapy, immunotherapy and surgical interventions. Drug therapy is usually the preferred initial approach; however, for patients with moderate to severe AR, it often fails to provide satisfactory results (Zaręba et al., 2024). Conversely, surgical treatment is a viable option for patients with AR when drug therapy is ineffective, particularly for those with concurrent nasal structural abnormalities (Ankermann and Brehler, 2023).

Vidian neurectomy (VN), a targeted surgical method, proposes severing the vidian nerve to mitigate autonomic responses that exacerbate AR symptoms (Chu, 2022). The disruption of the vidian nerve is thought to reduce autonomic hyperactivity, thereby decreasing glandular secretion and vascular congestion in the nasal mucosa. This surgical intervention is believed to alleviate the chronic symptoms of AR by directly modifying the underlying autonomic control mechanisms (Niu et al., 2024; Qi et al., 2021). Although the effectiveness of VN has been confirmed by multiple studies, the current evaluation of VN efficacy primarily relies on subjective symptom scores from patients, lacking objective indicators (Calderón et al., 2019).

In this study, we propose using nasal mucosal autonomic nerve responses, measured via an opisthenar sympathetic skin response (SSR) instrument (SSR-1000, BIOPAC, CA, United States), as an objective indicator of VN efficacy. Nasal mucosal autonomic nerve response refers to the reaction of the blood vessels and glands beneath the nasal mucosa when stimulated through the autonomic nervous system (Glatte et al., 2019). Electrophysiological recordings of this response can be conducted using the opisthenar SSR instrument. This instrument measures changes in skin resistance triggered by skin stimulation (Goizueta-San Martín et al., 2013), reflecting the activity level of the autonomic nervous system. By comparing these responses before and after VN, this study aims to validate the utility of this measurement as a reliable indicator of surgical success for patients with severe AR and provide a more quantitative assessment of VN’s impact on AR symptoms (Lin et al., 2021; Kanjanawasee and Tantilipikorn, 2022).

## 2 Materials and methods

### 2.1 Study participants

**Figure 1:**
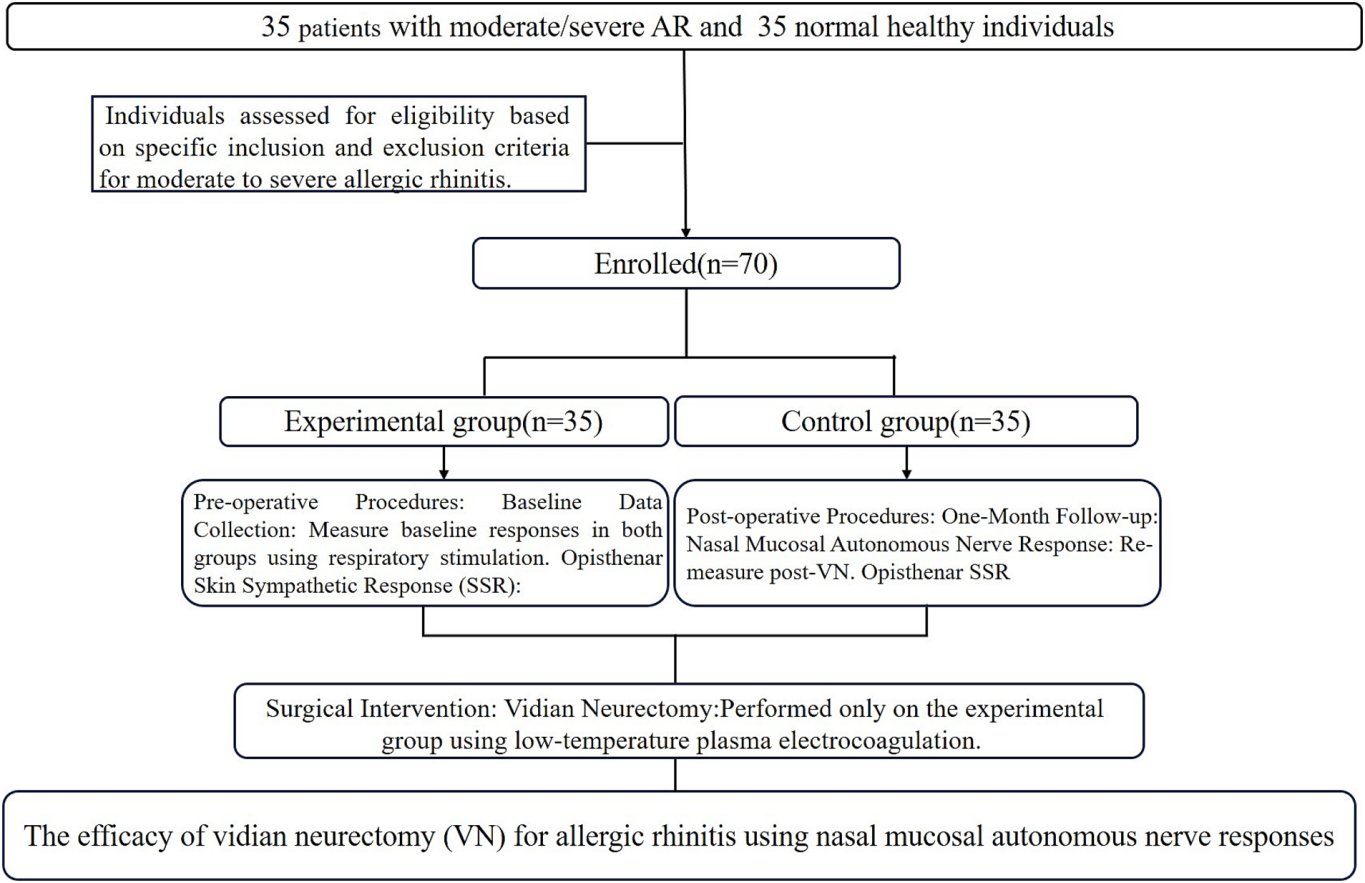
The flow diagram chart for this study.

This study was conducted in the Department of Otolaryngology between January 2023 and December 2023. The study obtained approval from the ethics committee of the hospital [Approval number: (2022) Ethical Review No. 061], and all participants signed informed consent forms. The study participants were divided into two groups: the experimental group (*n* = 35) and the control group (*n* = 35), as depicted in Figure 1. The inclusion criteria in the experimental group were as follows: (1) patients diagnosed with moderate to severe AR without age limitation; (2) selection adhered to the ARIA guidelines (Bousquet et al., 2019), which require AR symptoms to persist for over 4 weeks, occurring more than 4 days per week and lasting longer than 4 h each day; and (3) candidates were either unresponsive to conventional drug treatments or exhibited intolerance. The exclusion criteria in the experimental group included patients (1) who had undergone any nasal surgeries within the 6 months prior to the study; (2) were pregnant or lactating; (3) who had prior medical history or conditions that could influence autonomic nerve function or inflammatory responses, such as severe cardiovascular diseases, neurological disorders or other types of rhinitis; (4) with a history of autoimmune disorders, such as rheumatoid arthritis; and (5) who had used medications affecting the autonomic nervous system within the last 3 months, such as beta-blockers or immunosuppressants.

A total of 35 healthy individuals, matched in age and gender with the experimental group, were selected as the control group, with no history of AR or other nasal disorders, recent upper respiratory tract infections or chronic systemic diseases that could impact the autonomic nervous system. Controls were excluded if they had substantial health conditions that could interfere with the study parameters or if they had used medications known to affect the autonomic nervous system, such as beta-blockers, within the last 3 months.

### 2.2 Electrocoagulation of the vidian nerve

Patients in the experimental group underwent electrocoagulation of the vidian nerve (Lee et al., 2009), utilising a low-temperature plasma knife (Coblator II, ArthroCare, United States). Under nasal endoscopy, the nasal mucosa was anaesthetised and vasoconstricted using a solution of 1% lidocaine with 1:100,000 epinephrine. Additionally, local infiltration anaesthesia was performed in the pterygopalatine fossa with a 0.5% lidocaine and 1:100,000 epinephrine solution. Under nasal endoscopy, the posterior portion of the middle turbinate was resected to create sufficient exposure of the sphenopalatine foramen and to identify the vidian canal. The vidian nerve was located at the base of the pterygoid process within the pterygopalatine fossa. Dissection was carefully performed to preserve adjacent structures, including the sphenopalatine artery and maxillary nerve, which were clearly identified and protected during the procedure. The vidian nerve was then carefully electrocoagulated and subsequently severed using a low-temperature plasma knife, ensuring cautious preservation of the surrounding sphenopalatine artery and maxillary nerve. Post-procedure, both nasal cavities were packed with expandable haemostatic sponges, which were removed 24 h post-surgery. The nasal mucosal autonomic nerve response and SSR were re-evaluated 1 month after the operation to assess the effects of the surgery.

### 2.3 Sympathetic skin response measurement

The nasal mucosal autonomic nerve response was induced through respiratory stimulation (Anggård, 1993). Electrical signals from the nasal mucosa and the back of the hand were recorded using the SSR instrument. The procedures were as follows. Electrode patches were applied to the left and right earlobes, and a homemade simple electrode pen was placed on the lateral wall of the patient’s nasal cavity, tightly against the lateral mucosa of the anterior portion of the inferior turbinate. The left earlobe recorded the left nasal mucosal electrode, whereas the right earlobe recorded the right nasal mucosal electrode. A reference electrode was placed on the palm of the hand, and the SSR electrode was placed on the back of the hand. The SSR instrument was then connected, and the gain and filter were adjusted to ensure clear and stable signal reception. The respiratory stimulation protocol was standardised across all participants: inhaling room-temperature air for 5 min, followed by cold air at 10°C for another 5 min, then returning to room-temperature air for 5 min and finally inhaling hot air at 40°C for 5 min. The flow rate for each air type was maintained at 10 L/min, with temperature and humidity monitored using a thermometer-hygrometer. The electrical signals from the nasal mucosa and the back of the hand were recorded for each participant, analysing and measuring waveforms and amplitudes in millivolts (mV). The duration of the analysis was 750 ms and the sensitivity was 2.0 mV/Div. Each participant underwent the respiratory stimulation three times, and the average value from these three trials was taken as the experimental value. Waveforms were classified into P-type (positive wave), N-type (negative wave) and M-type (bidirectional wave), with the amplitude measured from the peak to the baseline, taking the maximum value as a reference.

### 2.4 Statistical analysis

For the statistical analysis of the data, SPSS 22.0 software (IBM, Armonk, NY, United States) was employed. A normality test was performed using the Shapiro–Wilk method. Measurement data were presented as mean ± standard deviation (x ± s), and count data as frequency (n) and percentage (%). The amplitudes of the nasal mucosal autonomic nerve response and the opisthenar SSR were compared between the experimental and control groups using the rank sum test or paired rank sum test, depending on the data distribution. A *p*-value of <0.05 was considered to indicate statistical significance.

## 3 Results

### 3.1 Waveform analysis of nasal mucosal autonomic responses and sympathetic skin response

**Figure 2:**
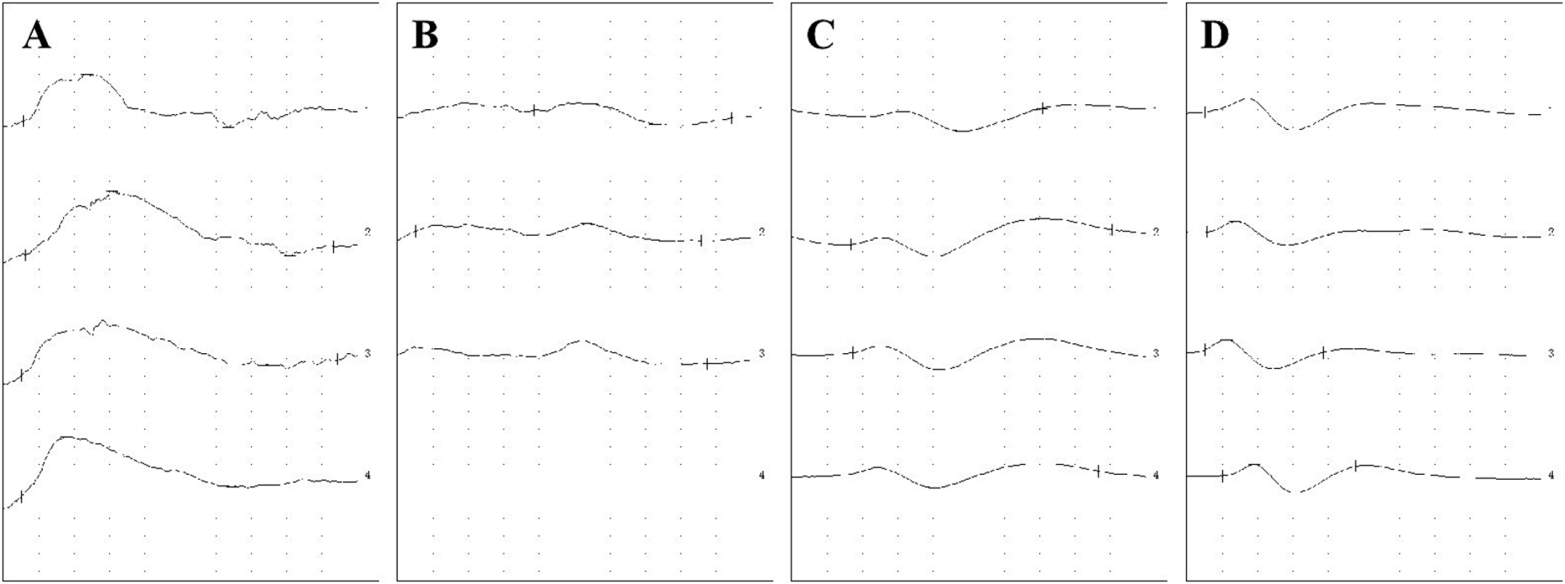
Waveforms of nasal autonomous nerve response and SSR **(A)** P-type of nasal autonomous nerve response. **(B)** N-type of nasal autonomous nerve response. **(C)** M-type of nasal autonomous nerve response. **(D)** M-type of SSR.

In this study, three types of waveforms were identified in the nasal mucosal autonomic nerve responses: P-type, N-type and M-type (Figure 2). The P-type waveform was most common, present in 23 of the 35 patients in the experimental group and 25 of the 35 in the control group. The N-type waveform was observed in 8 patients and the M-type in 4 patients within the experimental group.

The mean amplitude of the nasal mucosal autonomic nerve response in the experimental group before surgery was 3.52 ± 0.87 mV for the left nasal mucosa and 3.36 ± 0.83 mV for the right nasal mucosa. These values were significantly higher compared with those of the control group (*p* = 0.024 and 0.015). Postoperatively, the experimental group showed a decrease in amplitude to 1.18 ± 0.31 mV for the left and 0.83 ± 0.26 mV for the right nasal mucosa, which was significantly lower than the preoperative values (*p* < 0.001). However, when these postoperative amplitudes were compared with those of the control group, there was no statistically significant difference (left nasal mucosa: *p* = 0.492; right nasal mucosa: *p* = 0.357), as shown in Table 1. This suggests that VN surgery effectively reduced the nasal mucosal autonomic nerve response to levels comparable with those of the control group.

**Table 1:** Comparison of amplitudes of nasal mucosal autonomous response and opisthenar SSR between the experimental and control groups (x ± s, mV).

Group	Left nasal mucosa	Right nasal mucosa	Opisthenar
Experimental (Pre-surgery)	3.519 ± 0.872	3.360 ± 0.836	2.160 ± 0.524
Experimental (Post-surgery)	1.179 ± 0.312	0.829 ± 0.264	1.688 ± 0.412
Control	1.216 ± 0.328	1.072 ± 0.296	1.704 ± 0.416
Experimental (Pre-surgery) vs. Control	*Z* = −2.252, *p* = 0.024	*Z* = −2.421, *p* = 0.015	*Z* = −0.83, *p* = 0.407
Experimental (Post-surgery) vs. Control	*Z* = −0.687, *p* = 0.492	*Z* = −0.921，*p* = 0.357	*Z* = −0.121, *p* = 0.904
Experimental (Pre-surgery vs. Post-surgery)	*Z* = −4.013, *p* < 0.001	*Z* = −4.095, *p* < 0.001	*Z* = −2.408, *p* = 0.016

### 3.2 Analysis of the effect of comorbidities on surgery efficacy

Table 2 details the impact of specific comorbidities – nasal polyps, sinusitis and a deviated nasal septum – on surgical efficacy in the experimental group. There was no significant difference in the preoperative amplitude of autonomic nerve responses between the left and right nasal mucosa for these three comorbidities (*p* > 0.05). However, the mean postoperative amplitude of autonomic nerve responses in the left and right nasal mucosa was lower than the preoperative values for the three comorbidities, with statistically significant differences (left nasal mucosa: *p* = 0.002, <0.001 and 0.019; right nasal mucosa: *p* = 0.012, <0.001 and 0.006). Additionally, the postoperative amplitude of opisthenar SSR in the sinusitis and deviated nasal septum groups decreased compared with their preoperative values, and this reduction was statistically significant (*p* < 0.001 and *p* = 0.002). In contrast, no significant changes were observed in the preoperative and postoperative amplitudes of opisthenar SSR in the nasal polyp group, with these differences not reaching statistical significance (*p* = 0.578), as shown in Table 2.

**Table 2:** Effect of comorbidities on surgical efficacy in the experimental group (x ± s, mV).

Comorbidities	Left nasal mucosa	Right nasal mucosa	Opisthenar
Nasal polyps (Pre-surgery)	3.476 ± 0.864	3.312 ± 0.816	2.184 ± 0.536
Nasal polyps (Post-surgery)	1.192 ± 0.328	0.840 ± 0.272	1.712 ± 0.424
Sinusitis (Pre-surgery)	3.520 ± 0.896	3.360 ± 0.864	2.160 ± 0.528
Sinusitis (Post-surgery)	1.176 ± 0.304	0.824 ± 0.256	1.680 ± 0.408
Deviated nasal septum (Pre-surgery)	3.536 ± 0.832	3.424 ± 0.816	2.144 ± 0.512
Deviated nasal septum (Post-surgery)	1.168 ± 0.328	0.816 ± 0.264	1.664 ± 0.408
Nasal polyps (Pre-surgery) vs. Sinusitis (Pre-surgery)	*Z* = −0.147, *p* = 0.883	*Z* = −0.687, *p* = 0.492	*Z* = −1.146, *p* = 0.252
Nasal polyps (Pre-surgery) vs. Deviated nasal septum (Pre-surgery)	*Z* = −0.241, *p* = 0.810	*Z* = −0.361, *p* = 0.718	*Z* = −0.121, *p* = 0.904
Sinusitis (Pre-surgery) vs. Deviated nasal septum (Pre-surgery)	*Z* = −0.672, *p* = 0.543	*Z* = −1.258, *p* = 0.246	*Z* = −0.901, *p* = 0.375
Nasal polyps (Pre-surgery vs. Post-surgery)	Z = −3.076, *p* = 0.002	Z = −2.52, *p* = 0.012	Z = −0.676, *p* = 0.578
Sinusitis (Pre-surgery vs. Post-surgery)	*Z* = −4.042, *p* < 0.001	*Z* = −3.713, *p* < 0.001	*Z* = −4.042, *p* < 0.001
Deviated nasal septum (Pre-surgery vs. Post-surgery)	*Z* = −2.354, *p* = 0.019	*Z* = −2.731, *p* = 0.006	*Z* = −3.076, *p* = 0.002

## 4 Discussion

Allergic rhinitis represents a prevalent inflammatory disorder of the nasal cavity, the pathogenesis of which is closely linked to autonomic nervous system dysfunction (Chesné et al., 2019). While the therapeutic efficacy of VN has been substantiated by numerous studies, current assessment methods predominantly depend on patients’ subjective symptom scores, lacking robust objective evaluation parameters. This study demonstrates that nasal mucosal autonomic nerve response assessment can serve as a reliable method for evaluating the surgical outcomes of VN in AR treatment. This novel approach offers significant advantages in terms of simplicity, objectivity and reliability. Moreover, the presence of comorbid conditions, including nasal polyps, sinusitis and nasal septum deviation, appears to have no significant impact on the therapeutic effectiveness of VN in patients with AR.

The autonomic nervous system, which is not under conscious control, primarily includes the sympathetic and parasympathetic nervous systems. These regulate the activity of organs such as the viscera, blood vessels and glands in the human body (Rao, 2023). The activity level of the autonomic nervous system can be reflected by the SSR, which measures changes in skin resistance caused by stimulation through the autonomic nervous system, reflecting the excitability and sensitivity of this system (Lin et al., 2021). The waveform of the SSR is generally M-type, characterised by a positive wave followed by a negative wave, with the amplitude measured in mV as the distance between the peak and the baseline (Salameh et al., 2022).

In this study, three types of waveforms were identified in nasal mucosal autonomic nerve response. In contrast, only an M-type wave was found in the opisthenar SSR (Kamel et al., 2023). This indicates that the waveforms of the nasal mucosal autonomic nerve response are not entirely consistent with those of the opisthenar SSR, which may be attributed to the structural and functional differences between the autonomic nervous systems in the nasal mucosa and the back of the hand. The autonomic nervous system in the nasal mucosa primarily comprises the vidian nerve – a mixed nerve containing sensory, motor and parasympathetic fibres – and the nasal nerve, a purely sensory nerve containing sensory and sympathetic fibres (Senanayake et al., 2022). Conversely, the autonomic nervous system in the back of the hand primarily consists of the sympathetic nerve – a purely motor nerve containing motor and sympathetic nerve fibres – and the somatic nerve, a mixed nerve containing sensory, motor and parasympathetic nerve fibres (Min et al., 2017).

Although we acknowledge the difference in autonomic response waveforms between these two sites, the back of the hand serves as a baseline for comparing the localised effects of VN on the nasal mucosa. Given that the nasal mucosal response is more directly impacted by the vidian nerve, the comparison with the SSR of the back of the hand helps isolate the localised effect of the surgery on nasal autonomic function, rather than on the overall autonomic nervous system.

This study also found that preoperative amplitudes of the autonomic nerve response in the left and right nasal mucosa in the experimental group were higher than those in the control group. This suggests that the sensitivity and excitability of the nasal mucosal autonomic nerve response in patients with AR are heightened compared with those in individuals without AR, potentially due to nasal mucosal inflammation and allergic reactions. Upon allergen exposure, the nasal mucosa of patients with AR generates numerous inflammatory mediators, such as histamine, leukotrienes and prostaglandins. These mediators may activate or enhance the function of the nasal mucosal autonomic nervous system (Cavaliere et al., 2021). Consequently, the amplitude of the nasal mucosal autonomic nerve response can indicate the degree of nasal mucosal inflammation and allergic reaction in patients with AR.

The VN procedure was performed using a low-temperature plasma knife, which is a commonly used and effective tool for this surgery. Anatomical variations in the pterygopalatine fossa were carefully considered during the procedure to ensure the precision and safety of the nerve dissection. The VN procedure, which involves severing or electrocoagulating the vidian nerve, aims to alleviate AR symptoms by disrupting the nerve supply to the blood vessels and glands under the nasal mucosa (Calderón et al., 2019). Post-VN, damage or disruption to the parasympathetic nerve fibres in the nasal mucosa leads to constriction of the nasal submucosal blood vessels and diminished glandular secretion, thus alleviating symptoms such as nasal congestion, rhinorrhoea, sneezing and nasal itching (Hamilton et al., 2020). Furthermore, this study observed that postoperative amplitudes of the autonomic nerve response in the left and right nasal mucosa in the experimental group were significantly lower than their preoperative values. Hence, the decrease in amplitude of the nasal mucosal autonomic nerve response reflects changes in nasal mucosal nerve function post-VN and can be utilised as a measure for assessing the effectiveness of surgery.

Simultaneously, this study monitored preoperative and postoperative changes in the amplitude of the opisthenar SSR in the experimental group. No significant changes were observed. This indicates that VN has little effect on the amplitude of the opisthenar SSR, which may be due to anatomical and functional disparities between the autonomic nervous systems in the back of the hand and the nasal mucosa. As previously discussed, the nasal mucosal autonomic nervous system primarily comprises the vidian and intranasal nerves, whereas the autonomic nervous system in the back of the hand predominantly consists of the sympathetic and somatic nerves. These differences in composition and proportion may result in varying response modes and intensities to stimulation. Furthermore, the neural connections of the autonomic nervous system in the back of the hand differ from those in the nasal mucosa. The former is primarily connected to the central nervous system through the spinal cord and sympathetic ganglia, whereas the latter is connected through the trigeminal nerve and the pterygopalatine ganglion (Murrison et al., 2019). Consequently, the effects of VN on the autonomic nervous system of the nasal mucosa are unlikely to be transmitted to the autonomic nervous system in the back of the hand, leading to no substantial changes in the amplitude of the opisthenar SSR.

Additionally, an analysis was conducted on the effect of comorbidities such as nasal polyps, sinusitis and deviated nasal septum on the surgical efficacy in the experimental group. This analysis found no significant preoperative differences in the amplitudes of the autonomic nerve response in the left and right nasal mucosa for the three comorbidities. This suggests that the presence of nasal polyps, sinusitis and deviated nasal septum does not substantially affect the sensitivity and excitability of the nasal mucosal autonomic nerve responses in patients with AR, potentially due to the limited impact of these comorbidities on inflammation and allergic reactions in the nasal mucosa. Conversely, the average postoperative amplitude of the autonomic nerve response in the left and right nasal mucosa decreased compared with preoperative values across all three comorbidities. This indicates that the efficacy of VN in patients with AR and these comorbidities is consistent, likely due to the comprehensive blocking effect of VN on the nerve function of the nasal mucosa. However, postoperative opisthenar SSR amplitudes decreased for sinusitis and deviated septum groups but not for patients with nasal polyps. In the sinusitis and deviated septum groups, nasal airflow was less restricted, allowing for more consistent respiratory stimulation and a decrease in SSR amplitude postoperatively. In contrast, patients with nasal polyps had poorer nasal ventilation due to swelling and obstruction, resulting in weaker inspiratory stimuli and a smaller SSR response. Additionally, the more pronounced mucosal edema in patients with nasal polyps may have affected the SSR peak or amplitude, explaining the lack of significant postoperative change in this group.

This study is limited by several factors. First, the relatively small sample size and lack of diversity may limit the generalisability of the findings. Second, the follow-up was short-term, and it is currently unclear whether amplitude reductions can serve as predictive markers for long-term symptom control. Given the complexity of AR and the potential for nerve reinnervation or other long-term factors, future studies with extended follow-up periods are needed to assess the long-term correlation between amplitude reductions and sustained symptom relief. Third, the study lacked comparative analysis, and without a comparison to another intervention, it is challenging to determine the relative efficacy of VN. Additionally, the lack of patient-reported subjective symptom scores restricts the ability to correlate these objective findings with clinical improvements in patients’ symptoms. Future studies should incorporate both objective and subjective measures to more comprehensively evaluate the clinical efficacy of VN. Finally, the method of autonomic nerve response monitoring of the nasal mucosa is a novel approach. Further validation and comparison with traditional methods are needed to assess its reliability and precision using larger and more diverse patient populations.

## Data Availability

The original contributions presented in the study are included in the article/supplementary material, further inquiries can be directed to the corresponding author.
